# Bis(*N*,*N*-diisopropyl­butanaminium) bis­[di-μ-chlorido-bis­[dichlorido­cuprate(II)]]

**DOI:** 10.1107/S1600536810054280

**Published:** 2011-01-15

**Authors:** Jing Dai, Jie Xu

**Affiliations:** a Ordered Matter Science Research Center, College of Chemistry and Chemical Engineering, Southeast University, Nanjing 210096, People’s Republic of China

## Abstract

In the title compound, (C_10_H_24_N)_2_[Cu_2_Cl_6_], *N*,*N*-diisopropyl­butanamine is protonated on the N atom. The Cu^II^ atom in the centrosymmetric [Cu_2_Cl_6_]^2−^ anion has a distorted tetra­hedral geometry. In the crystal, the cations and anions are connected by N—H⋯Cl and C—H⋯Cl hydrogen bonds into layers parallel to (100).

## Related literature

For the properties and structures of *N*,*N*-diisopropyl­butyl-1-amine compounds, see: Fu *et al.* (2007 ▶, 2008 ▶, 2009 ▶); Fu & Xiong (2008 ▶).
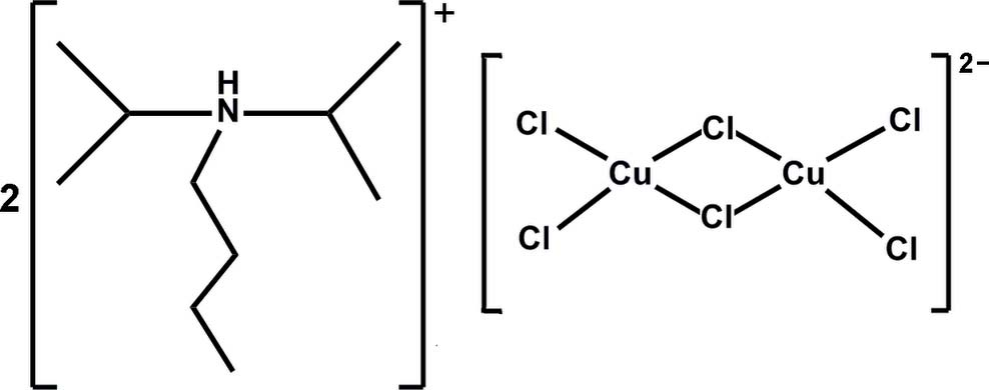


## Experimental

### 

#### Crystal data

(C_10_H_24_N)_2_[Cu_2_Cl_6_]
*M* *_r_* = 656.40
Triclinic, 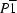

*a* = 8.5697 (17) Å
*b* = 10.213 (2) Å
*c* = 10.384 (2) Å
α = 72.43 (3)°
β = 68.53 (3)°
γ = 71.78 (3)°
*V* = 785.1 (3) Å^3^
*Z* = 1
Mo *K*α radiation
μ = 1.88 mm^−1^
*T* = 298 K
0.30 × 0.05 × 0.05 mm

#### Data collection

Rigaku Mercury2 CCD diffractometer
Absorption correction: multi-scan (*CrystalClear*; Rigaku, 2005 ▶) *T*_min_ = 0.910, *T*_max_ = 1.000
8206 measured reflections
3588 independent reflections
2728 reflections with *I* > 2σ(*I*)
*R*_int_ = 0.044

#### Refinement

*R*[*F* ^2^ > 2σ(*F* ^2^)] = 0.046
*wR*(*F* ^2^) = 0.107
*S* = 1.08
3588 reflections
141 parameters
H-atom parameters constrained
Δρ_max_ = 0.45 e Å^−3^
Δρ_min_ = −0.52 e Å^−3^

### 

Data collection: *CrystalClear* (Rigaku, 2005 ▶); cell refinement: *CrystalClear*; data reduction: *CrystalClear*; program(s) used to solve structure: *SHELXS97* (Sheldrick, 2008 ▶); program(s) used to refine structure: *SHELXL97* (Sheldrick, 2008 ▶); molecular graphics: *SHELXTL* (Sheldrick, 2008 ▶) and *Mercury* (Macrae *et al.*, 2006 ▶); software used to prepare material for publication: *SHELXTL*.

## Supplementary Material

Crystal structure: contains datablocks I, global. DOI: 10.1107/S1600536810054280/hy2392sup1.cif

Structure factors: contains datablocks I. DOI: 10.1107/S1600536810054280/hy2392Isup2.hkl

Additional supplementary materials:  crystallographic information; 3D view; checkCIF report

## Figures and Tables

**Table 1:** Hydrogen-bond geometry (Å, °)

*D*—H⋯*A*	*D*—H	H⋯*A*	*D*⋯*A*	*D*—H⋯*A*
N1—H1⋯Cl3	0.91	2.44	3.336 (3)	168
C2—H2*A*⋯Cl3^i^	0.98	2.82	3.668 (5)	146
C5—H5*A*⋯Cl2^ii^	0.98	2.64	3.511 (4)	149
C8—H8*A*⋯Cl3	0.97	2.78	3.617 (4)	144

Symmetry codes: (i) 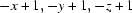; (ii) 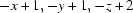.

**Fig. 1.**
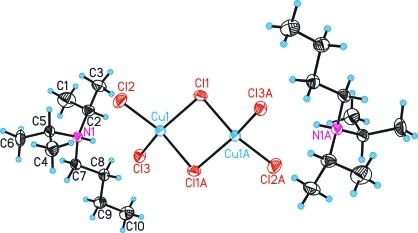
Molecular structure of the title compound. Displacement ellipsoids are drawn at the 30% probability level. [Symmetry code: (A) 1-x, 2-y, 1-z.]

**Fig. 2.**
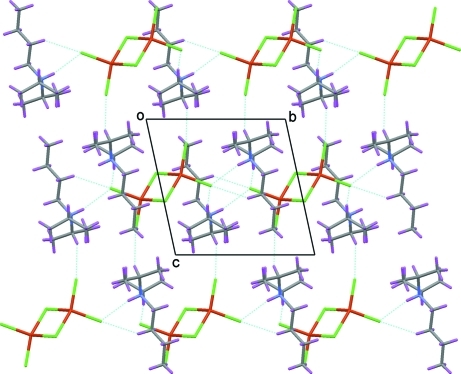
The crystal packing of the title compound, showing the two-dimensional network. Dashed lines denote hydrogen bonds.

## supplementary crystallographic information

### Comment

Salts of amide have attracted more attention as phase transition dielectric materials for its applications in memory storage (Fu *et al.*, 2007, 2008, 2009; Fu & Xiong, 2008). With the purpose of obtaining phase transition crystals of *N*,*N*-diisopropylbutyl-1-amine salts, its interactions with various metal ions have been studied and we have elaborated a series of new materials with this organic molecule. In this paper, we describe the crystal structure of the title compound,

The asymmetric unit is composed of half [Cu_2_Cl_6_]^2-^ anion and one [C_10_H_24_N]^+^ cation (Fig. 1). The N atom of *N*,*N*-diisopropylbutyl-1-amine is protonated, thus indicating one positive charge in the amide N atom. The half [Cu_2_Cl_6_]^2-^ anion showing one negative charge makes charge balance. The geometric parameters of the title compound are in a normal range. In the crystal structure, the cations and anions are involved in N—H···Cl and C—H···Cl hydrogen bonds (Table 1), which link the cations and anions into a two-dimensional network (Fig. 2).

### Experimental

The commercial *N*,*N*-diisopropylbutyl-1-amine (10 mmol), HCl (2 ml) and CuCl_2_ (5 mmol) were dissolved in 10 ml water. The solvent was slowly evaporated in air, affording blue block-shaped crystals of the title compound suitable for X-ray analysis.

The dielectric constant of the title compound as a function of temperature indicates that the permittivity is basically temperature-independent, suggesting that this compound should not be a real ferroelectrics or there may be no distinct phase transition occurred within the measured temperature range. Similarly, below the melting point (446–447 K) of the compound, the dielectric constant as a function of temperature also goes smoothly, and there is no dielectric anomaly observed (dielectric constant equaling to 8.9 to 10.6).

### Refinement

H atoms were positioned geometrically and treated as riding, with C—H = 0.98 (CH), 0.97 (CH_2_) and 0.96 (CH_3_) Å and N—H = 0.91 Å and with *U*_iso_(H) = 1.2(1.5 for methyl)*U*_eq_(C, N).

### Crystal data

**Table:**

(C_10_H_24_N)_2_[Cu_2_Cl_6_]	*Z* = 1
*M**_r_* = 656.40	*F*(000) = 342
Triclinic, *P*1	*D*_x_ = 1.388 Mg m^−^^3^
Hall symbol: -P 1	Mo *K*α radiation, λ = 0.71073 Å
*a* = 8.5697 (17) Å	Cell parameters from 3588 reflections
*b* = 10.213 (2) Å	θ = 3.4–27.5°
*c* = 10.384 (2) Å	µ = 1.88 mm^−^^1^
α = 72.43 (3)°	*T* = 298 K
β = 68.53 (3)°	Block, blue
γ = 71.78 (3)°	0.30 × 0.05 × 0.05 mm
*V* = 785.1 (3) Å^3^	

### Data collection

**Table:**

Rigaku Mercury2 CCD diffractometer	3588 independent reflections
Radiation source: fine-focus sealed tube	2728 reflections with *I* > 2σ(*I*)
graphite	*R*_int_ = 0.044
Detector resolution: 13.6612 pixels mm^-1^	θ_max_ = 27.5°, θ_min_ = 3.4°
ω scans	*h* = −11→11
Absorption correction: multi-scan (*CrystalClear*; Rigaku, 2005)	*k* = −13→13
*T*_min_ = 0.910, *T*_max_ = 1.000	*l* = −13→13
8206 measured reflections	

### Refinement

**Table:**

Refinement on *F*^2^	Primary atom site location: structure-invariant direct methods
Least-squares matrix: full	Secondary atom site location: difference Fourier map
*R*[*F*^2^ > 2σ(*F*^2^)] = 0.046	Hydrogen site location: inferred from neighbouring sites
*wR*(*F*^2^) = 0.107	H-atom parameters constrained
*S* = 1.08	*w* = 1/[σ^2^(*F*_o_^2^) + (0.0342*P*)^2^ + 0.4528*P*] where *P* = (*F*_o_^2^ + 2*F*_c_^2^)/3
3588 reflections	(Δ/σ)_max_ < 0.001
141 parameters	Δρ_max_ = 0.45 e Å^−^^3^
0 restraints	Δρ_min_ = −0.52 e Å^−^^3^

### Fractional atomic coordinates and isotropic or equivalent isotropic displacement parameters (Å^2^)

**Table:**

	*x*	*y*	*z*	*U*_iso_*/*U*_eq_	
N1	0.7275 (3)	0.3131 (3)	0.7043 (3)	0.0374 (6)	
H1	0.7197	0.4066	0.6644	0.045*	
C1	0.5308 (7)	0.1423 (5)	0.8060 (6)	0.0907 (17)	
H1A	0.4248	0.1297	0.8051	0.136*	
H1B	0.5300	0.1267	0.9021	0.136*	
H1C	0.6256	0.0761	0.7589	0.136*	
Cu1	0.55821 (5)	0.83754 (4)	0.59096 (4)	0.03888 (14)	
Cl1	0.30886 (11)	1.01180 (9)	0.60170 (11)	0.0629 (3)	
C2	0.5495 (4)	0.2903 (4)	0.7304 (4)	0.0510 (9)	
H2A	0.5344	0.3062	0.6374	0.061*	
Cl2	0.50922 (16)	0.75177 (13)	0.81673 (10)	0.0778 (4)	
C3	0.4106 (5)	0.3995 (5)	0.8062 (5)	0.0680 (12)	
H3A	0.3011	0.4003	0.8002	0.102*	
H3B	0.4357	0.4909	0.7625	0.102*	
H3C	0.4069	0.3766	0.9038	0.102*	
Cl3	0.70329 (12)	0.64171 (9)	0.51116 (9)	0.0505 (2)	
C4	0.8746 (6)	0.4013 (5)	0.8158 (4)	0.0663 (12)	
H4A	0.8994	0.3922	0.9015	0.099*	
H4B	0.8065	0.4941	0.7912	0.099*	
H4C	0.9805	0.3864	0.7407	0.099*	
C5	0.7756 (4)	0.2922 (4)	0.8379 (4)	0.0449 (8)	
H5A	0.6679	0.3114	0.9140	0.054*	
C6	0.8723 (6)	0.1434 (4)	0.8862 (4)	0.0707 (13)	
H6A	0.8802	0.1331	0.9787	0.106*	
H6B	0.9860	0.1261	0.8209	0.106*	
H6C	0.8119	0.0771	0.8894	0.106*	
C7	0.8673 (4)	0.2391 (4)	0.5948 (3)	0.0447 (8)	
H7A	0.8709	0.1389	0.6258	0.054*	
H7B	0.9768	0.2520	0.5902	0.054*	
C8	0.8484 (5)	0.2876 (4)	0.4479 (3)	0.0452 (8)	
H8A	0.8366	0.3890	0.4183	0.054*	
H8B	0.7446	0.2670	0.4494	0.054*	
C9	1.0015 (5)	0.2162 (4)	0.3426 (4)	0.0565 (10)	
H9A	1.1046	0.2375	0.3417	0.068*	
H9B	1.0137	0.1149	0.3740	0.068*	
C10	0.9891 (6)	0.2596 (5)	0.1937 (4)	0.0698 (12)	
H10A	1.0904	0.2107	0.1328	0.105*	
H10B	0.9796	0.3595	0.1607	0.105*	
H10C	0.8893	0.2364	0.1929	0.105*	

### Atomic displacement parameters (Å^2^)

**Table:**

	*U*^11^	*U*^22^	*U*^33^	*U*^12^	*U*^13^	*U*^23^
N1	0.0384 (15)	0.0359 (14)	0.0347 (15)	−0.0041 (11)	−0.0109 (12)	−0.0078 (12)
C1	0.083 (3)	0.072 (3)	0.124 (5)	−0.045 (3)	−0.018 (3)	−0.016 (3)
Cu1	0.0400 (2)	0.0323 (2)	0.0370 (2)	−0.00387 (16)	−0.00747 (17)	−0.00665 (17)
Cl1	0.0393 (5)	0.0424 (5)	0.0699 (7)	−0.0006 (4)	0.0065 (4)	0.0027 (4)
C2	0.043 (2)	0.066 (2)	0.047 (2)	−0.0150 (18)	−0.0124 (17)	−0.0148 (19)
Cl2	0.0893 (8)	0.0808 (8)	0.0353 (5)	−0.0012 (6)	−0.0073 (5)	−0.0061 (5)
C3	0.040 (2)	0.084 (3)	0.070 (3)	−0.007 (2)	−0.009 (2)	−0.019 (2)
Cl3	0.0628 (6)	0.0332 (4)	0.0488 (5)	−0.0008 (4)	−0.0151 (4)	−0.0118 (4)
C4	0.073 (3)	0.083 (3)	0.058 (3)	−0.023 (2)	−0.031 (2)	−0.017 (2)
C5	0.0412 (19)	0.053 (2)	0.0355 (19)	−0.0014 (16)	−0.0136 (15)	−0.0097 (16)
C6	0.084 (3)	0.065 (3)	0.054 (3)	0.011 (2)	−0.039 (2)	−0.005 (2)
C7	0.0429 (19)	0.0426 (19)	0.042 (2)	0.0019 (15)	−0.0118 (16)	−0.0134 (16)
C8	0.051 (2)	0.0408 (19)	0.044 (2)	−0.0037 (16)	−0.0172 (17)	−0.0138 (16)
C9	0.057 (2)	0.062 (2)	0.046 (2)	−0.0047 (19)	−0.0102 (18)	−0.0212 (19)
C10	0.081 (3)	0.083 (3)	0.049 (2)	−0.017 (2)	−0.012 (2)	−0.030 (2)

### Geometric parameters (Å, °)

**Table:**

N1—C7	1.501 (4)	C4—H4B	0.9600
N1—C5	1.525 (4)	C4—H4C	0.9600
N1—C2	1.528 (4)	C5—C6	1.518 (5)
N1—H1	0.9100	C5—H5A	0.9800
C1—C2	1.506 (5)	C6—H6A	0.9600
C1—H1A	0.9600	C6—H6B	0.9600
C1—H1B	0.9600	C6—H6C	0.9600
C1—H1C	0.9600	C7—C8	1.508 (4)
Cu1—Cl2	2.1701 (13)	C7—H7A	0.9700
Cu1—Cl3	2.2190 (12)	C7—H7B	0.9700
Cu1—Cl1^i^	2.2884 (14)	C8—C9	1.511 (5)
Cu1—Cl1	2.3051 (13)	C8—H8A	0.9700
Cl1—Cu1^i^	2.2884 (14)	C8—H8B	0.9700
C2—C3	1.515 (5)	C9—C10	1.509 (5)
C2—H2A	0.9800	C9—H9A	0.9700
C3—H3A	0.9600	C9—H9B	0.9700
C3—H3B	0.9600	C10—H10A	0.9600
C3—H3C	0.9600	C10—H10B	0.9600
C4—C5	1.519 (5)	C10—H10C	0.9600
C4—H4A	0.9600		
			
C7—N1—C5	113.3 (2)	C6—C5—C4	111.9 (3)
C7—N1—C2	113.5 (3)	C6—C5—N1	113.9 (3)
C5—N1—C2	114.8 (3)	C4—C5—N1	109.1 (3)
C7—N1—H1	104.6	C6—C5—H5A	107.2
C5—N1—H1	104.6	C4—C5—H5A	107.2
C2—N1—H1	104.6	N1—C5—H5A	107.2
C2—C1—H1A	109.5	C5—C6—H6A	109.5
C2—C1—H1B	109.5	C5—C6—H6B	109.5
H1A—C1—H1B	109.5	H6A—C6—H6B	109.5
C2—C1—H1C	109.5	C5—C6—H6C	109.5
H1A—C1—H1C	109.5	H6A—C6—H6C	109.5
H1B—C1—H1C	109.5	H6B—C6—H6C	109.5
Cl2—Cu1—Cl3	99.48 (5)	N1—C7—C8	115.2 (3)
Cl2—Cu1—Cl1^i^	146.21 (6)	N1—C7—H7A	108.5
Cl3—Cu1—Cl1^i^	96.22 (5)	C8—C7—H7A	108.5
Cl2—Cu1—Cl1	97.69 (6)	N1—C7—H7B	108.5
Cl3—Cu1—Cl1	143.17 (5)	C8—C7—H7B	108.5
Cl1^i^—Cu1—Cl1	87.01 (5)	H7A—C7—H7B	107.5
Cu1^i^—Cl1—Cu1	92.99 (5)	C7—C8—C9	111.6 (3)
C1—C2—C3	112.0 (4)	C7—C8—H8A	109.3
C1—C2—N1	113.3 (3)	C9—C8—H8A	109.3
C3—C2—N1	110.3 (3)	C7—C8—H8B	109.3
C1—C2—H2A	107.0	C9—C8—H8B	109.3
C3—C2—H2A	107.0	H8A—C8—H8B	108.0
N1—C2—H2A	107.0	C10—C9—C8	114.0 (3)
C2—C3—H3A	109.5	C10—C9—H9A	108.8
C2—C3—H3B	109.5	C8—C9—H9A	108.8
H3A—C3—H3B	109.5	C10—C9—H9B	108.8
C2—C3—H3C	109.5	C8—C9—H9B	108.8
H3A—C3—H3C	109.5	H9A—C9—H9B	107.6
H3B—C3—H3C	109.5	C9—C10—H10A	109.5
C5—C4—H4A	109.5	C9—C10—H10B	109.5
C5—C4—H4B	109.5	H10A—C10—H10B	109.5
H4A—C4—H4B	109.5	C9—C10—H10C	109.5
C5—C4—H4C	109.5	H10A—C10—H10C	109.5
H4A—C4—H4C	109.5	H10B—C10—H10C	109.5
H4B—C4—H4C	109.5		
			
Cl2—Cu1—Cl1—Cu1^i^	146.38 (6)	C2—N1—C5—C6	92.1 (4)
Cl3—Cu1—Cl1—Cu1^i^	−96.39 (7)	C7—N1—C5—C4	85.3 (4)
Cl1^i^—Cu1—Cl1—Cu1^i^	0.0	C2—N1—C5—C4	−142.1 (3)
C7—N1—C2—C1	70.5 (4)	C5—N1—C7—C8	−163.7 (3)
C5—N1—C2—C1	−62.1 (4)	C2—N1—C7—C8	63.0 (4)
C7—N1—C2—C3	−163.1 (3)	N1—C7—C8—C9	175.3 (3)
C5—N1—C2—C3	64.3 (4)	C7—C8—C9—C10	179.4 (3)
C7—N1—C5—C6	−40.5 (4)		

Symmetry codes: (i) −*x*+1, −*y*+2, −*z*+1.

### Hydrogen-bond geometry (Å, °)

**Table:**

*D*—H···*A*	*D*—H	H···*A*	*D*···*A*	*D*—H···*A*
N1—H1···Cl3	0.91	2.44	3.336 (3)	168
C2—H2A···Cl3^ii^	0.98	2.82	3.668 (5)	146
C5—H5A···Cl2^iii^	0.98	2.64	3.511 (4)	149
C8—H8A···Cl3	0.97	2.78	3.617 (4)	144

Symmetry codes: (ii) −*x*+1, −*y*+1, −*z*+1; (iii) −*x*+1, −*y*+1, −*z*+2.

## References

[bb1] Fu, D.-W., Ge, J.-Z., Dai, J., Ye, H.-Y. & Qu, Z.-R. (2009). *Inorg. Chem. Commun.* **12**, 994–997.

[bb2] Fu, D.-W., Song, Y.-M., Wang, G.-X., Ye, Q., Xiong, R.-G., Akutagawa, T., Nakamura, T., Chan, P. W. H. & Huang, S. D. (2007). *J. Am. Chem. Soc.* **129**, 5346–5347. 10.1021/ja0701816 17428055

[bb3] Fu, D.-W. & Xiong, R.-G. (2008). *Dalton Trans.* pp. 3946–3948. 10.1039/b806255b 18648695

[bb4] Fu, D.-W., Zhang, W. & Xiong, R.-G. (2008). *Cryst. Growth Des.* **8**, 3461–3464.

[bb5] Macrae, C. F., Edgington, P. R., McCabe, P., Pidcock, E., Shields, G. P., Taylor, R., Towler, M. & van de Streek, J. (2006). *J. Appl. Cryst.* **39**, 453–457.

[bb6] Rigaku (2005). *CrystalClear* Rigaku Corporation, Tokyo, Japan.

[bb7] Sheldrick, G. M. (2008). *Acta Cryst.* A**64**, 112–122. 10.1107/S0108767307043930 18156677

